# Relative Bioavailability of Iron in Bangladeshi Traditional Meals Prepared with Iron-Fortified Lentil Dal

**DOI:** 10.3390/nu10030354

**Published:** 2018-03-15

**Authors:** Rajib Podder, Diane M. DellaValle, Robert T. Tyler, Raymond P. Glahn, Elad Tako, Albert Vandenberg

**Affiliations:** 1 Department of Plant Sciences, University of Saskatchewan, Saskatoon, SK S7N 5A8, Canada; rap039@mail.usask.ca; 2 Department of Nutrition and Dietetics, Marywood University, 2300 Adams Avenue, Scranton, PA 18509, USA; ddellavalle@maryu.marywood.edu; 3 Department of Food and Bioproduct Sciences, University of Saskatchewan, Saskatoon, SK S7N 5A8, Canada; bob.tyler@usask.ca; 4 Robert W. Holley Center for Agriculture and Health, Agricultural Research Service, USDA, Ithaca, NY 14853, USA; rpg3@cornell.edu (R.P.G.); et79@cornell.edu (E.T.)

**Keywords:** lentil, iron, fortification, bioavailability, Bangladesh

## Abstract

Due to low Fe bioavailability and low consumption per meal, lentil must be fortified to contribute significant bioavailable Fe in the Bangladeshi diet. Moreover, since red lentil is dehulled prior to consumption, an opportunity exists at this point to fortify lentil with Fe. Thus, in the present study, lentil was Fe-fortified (using a fortificant Fe concentration of 2800 µg g^−1^) and used in 30 traditional Bangladeshi meals with broad differences in concentrations of iron, phytic acid (PA), and relative Fe bioavailability (RFeB%). Fortification with NaFeEDTA increased the iron concentration in lentil from 60 to 439 µg g^−1^ and resulted in a 79% increase in the amount of available Fe as estimated by Caco-2 cell ferritin formation. Phytic acid levels were reduced from 6.2 to 4.6 mg g^−1^ when fortified lentil was added, thereby reducing the PA:Fe molar ratio from 8.8 to 0.9. This effect was presumably due to dephytinization of fortified lentil during the fortification process. A significant (*p* ≤ 0.01) Pearson correlation was observed between Fe concentration and RFeB% and between RFeB% and PA:Fe molar ratio in meals with fortified lentil, but not for the meal with unfortified lentil. In conclusion, fortified lentil can contribute significant bioavailable Fe to populations at risk of Fe deficiency.

## 1. Introduction

Iron (Fe) deficiency is a public health problem and more than 30% of the world population (two billion) is anaemic, mainly due to Fe deficiency [1]. Fe deficiency is considered the major cause of anaemia, which mostly affects young children and pregnant and post-partum women [2]. In Bangladesh, anaemia is a public health concern and 40% of adolescents are anaemic [3]. In 2011, the national prevalence of anaemia in Bangladesh was 51% in children aged 6–59 months and 42% in non-pregnant women [4]. One of the major causes of Fe deficiency is low bioavailability of dietary Fe, especially in developing countries such as Bangladesh where diets are mostly cereal- and legume-based [5].

Among legumes, lentil is one of the oldest and most important cultivated crops. Lentil is consumed in both developed and developing countries around the world, and is a potential whole food source that can provide micronutrients such as Fe, zinc (Zn), and selenium (Se) [6]. In some developing countries, lentil is considered a staple food due to its nutritive value, especially as an inexpensive protein source compared to animal protein. Studies investigating ways to increase Fe content and bioavailability have focused mainly on biofortification strategies using marker-assisted breeding, improved agronomic practices, and removal of the seed coat from lentil seed [7,8,9]. However, Fe biofortification of food crops has several drawbacks, such as low bioavailability, limitations to increasing the total content in food crops, and insufficient consumption to show significant health benefits. The bioavailability of Fe from lentil is often compromised due to the presence of antinutritional factors (e.g., phytate, polyphenols, cotyledon cell wall) in the seed [10,11]. Fortification, on the other hand, often can overcome the inhibitors and provide significant bioavailable Fe [12] as long as the addition of Fe does not alter the appearance and taste of the target food product.

The main objective of any fortification program is to improve nutrient content and the nutritional quality of the added nutrients and thus help to eliminate or prevent deficiencies in the target population. Different strategies have been adopted to combat micronutrient deficiencies, such as biofortification, fortification, supplementation, dietary diversification, and nutrition education [13]. All of these strategies have limitations depending on sociocultural and economic factors as well as the age and gender of the target population. These may be overcome by food fortification, which has proven to be a cost-effective way to add micronutrients to processed food and improve the dietary quality of a target population without changing their food habits [14]. A systematic review of intervention of micronutrient fortified food and its impact on women and child health revealed that fortification with micronutrients, including Fe, significantly increased serum Fe concentrations with no significant adverse effect on hemoglobin levels [15].

Biofortification of lentil is not likely to have an impact on much of the Bangladeshi population as the consumption rate of pulses for the population of Bangladesh is 12 g/day/person [16], which is far below the desirable intake of 50 g/day/person that has been reported on the basis of previous studies and the current consumption pattern of the Bangladeshi population [17]. To address this shortfall, improving the nutritional quality of lentil by Fe fortification could provide a significant amount of the required daily Fe from a minimum amount of lentil dal, without having to increase the quantity of lentil in a given meal. To enable this approach, we previously developed a laboratory-scale protocol for fortifying de-hulled lentil seed (dal) using three Fe fortificants. NaFeEDTA was the most effective; at a fortificant Fe concentration of 1600 µg g^−1^, NaFeEDTA provided 13–14 mg of additional Fe per 100 g of cooked lentil dal [18]. The United States Food and Drug Administration (FDA) published a food fortification policy featuring six principles for food fortification [19,20]. These are: “(1) the nutrient intake without fortification is below the desirable content for a significant portion of the population; (2) the food being fortified is consumed in quantities that would make a significant contribution to the population’s intake of the nutrient; (3) the additional nutrient intake resulting from fortification is unlikely to create an imbalance of essential nutrients; (4) the nutrient added is stable under proper conditions of storage and use; (5) the nutrient is physiologically available from the food to which it is being added; and (6) there is reasonable assurance that it will not result in potentially toxic intakes.” All of these principles have been considered with respect to lentil fortification.

We also investigated the sensory acceptability of fortified lentil dal with respect to appearance, odor, taste, texture, and overall acceptability by lentil consumers [21]. Fortification of lentil with NaFeEDTA minimally affected consumer perception of appearance, taste, texture, odour, and overall acceptability of cooked lentil compared to fortification with FeSO_4_·7H_2_O or FeSO_4_·H_2_O. Sensory acceptability was statistically similar to that of non-fortified lentil for almost all of the attributes.

The present study aimed to determine the concentration and relative bioavailability of Fe in different traditional Bangladeshi meal plan models featuring fortified and unfortified lentil dal. A Caco-2 cell bioassay was used to assess relative Fe bioavailability (RFeB%), expressed as a percentage of that of an unfortified control red lentil sample that was included in each run of the bioassay. This lentil sample had an Fe concentration of 50 µg g^−1^. Ferritin formation by Caco-2 cell monolayers is a sensitive and accurate measurement tool for in vitro assessment of Fe bioavailability in food [22]. The concentration of phytic acid (PA), a known inhibitor of Fe bioavailability, also was determined in the meal plan models.

## 2. Materials and Methods

### 2.1. Preparation of Meal Models 

A total of 30 meal combinations were prepared and assessed with respect to Fe concentration, RFeB%, and PA concentration (Table S1). Among these, models 1 to 11 and 15 to 25 featured either unfortified or fortified lentil dal, respectively, in different amounts (% by weight) along with other meal components. Three models (models 12 to 14) contained no lentil. The remaining five models (models 26 to 30) were prepared with only rice (model 26), vegetables (model 27), fish (model 28), unfortified cooked dal (model 29), or NaFeEDTA-fortified cooked dal (model 30). The fortified lentil had been treated with 2800 µg g^−1^ ppm NaFeEDTA, which in previous work comparing various fortificants and concentrations thereof, was determined to have the least effect on appearance and consumer acceptability measures such as taste and texture [18,21]. Lentil dal was prepared according to a traditional Bangladeshi recipe [23] where lentil, deionized water, canola oil, salt, turmeric powder and onion were used as ingredients in a 15:70:4:3:2:6 ratio, by weight. Along with the dal, rice (white, boiled and unenriched), vegetables (mixture of carrot, cauliflower, brinjal, potato, sweet potato, onion, salt, turmeric, garlic, oil, and water at a 10:10:8:10:5:2:1:1:1:12:40 ratio, by weight) and fish (fish fillets, salt, turmeric, and oil at a 90:2:3:5 ratio, by weight) were used in different ratios to prepare the meal models. All foods were cooked with 18 MΩ deionized water. Rice, fish, and vegetables were cooked in a traditional Bangladeshi fashion. Stainless steel cookware was used to prepare all meal components. Prepared dishes were cooled at room temperature for 2 h, frozen at −80 °C for 24 h, freeze-dried using a FreeZone 12 L Console Freeze Dry System with Stoppering Tray Dryers (Labconco, model 7759040, Prospect Avenue, Kansas City, MO, USA) for 72 h, and stored at room temperature [24]. A 10-g sample from each freeze-dried cooked dish (models 1 to 30) was finely ground and sent to the USDA-ARS Robert Holley Center for Agriculture and Health (Ithaca, NY, USA) to determine Fe concentration, phytic acid concentration, and RFeB%. From the 10-g sample, 0.5 g of each of the three repetitions was used in the Caco-2 cell bioassay to estimate the RFeB% [24,25].

### 2.2. Assessment of Fe Concentration, RFeB%, and PA Concentration 

The concentrations of Fe for the 30 meal models were quantified with an inductively coupled argon-plasma emission spectrometer (iCAP 6500 series, Thermo Jarrell Ash Corp., Franklin, MA, USA) following the procedure of Glahn et al. (2017) [26]. Ferric chloride (FeCl_3_) was used as the certified reference material in the iCAP analysis. Relative bioavailability of Fe for the 30 meal models was assessed using an established Caco-2 cell bioassay, where Caco-2 cell ferritin formation is used as the measure of cell Fe uptake and bioavailability [7,22,27]. The bioavailability assessment was conducted on three replicates for each cooked lentil sample. Ferritin values from the fortified lentil samples were compared with the control lentil (CDC Robin; Fe concentration of 50 μg g^−1^) to calculate the RFeB%, using the following equation: Relative Fe bioavailability (RFeB %) = ((ng ferritin of the lentil sample/mg protein of the lentil sample)/(ng ferritin/mg protein of the control lentil)) × 100 [8]. The resulting index of relative Fe bioavailability (RFeB%) is used hereafter. Phytic acid content was measured as phosphorous released by phytase and alkaline phosphatase via a colorimetric assay kit (K-PHYT 12/12, Megazyme International, Wicklow, Ireland) [26].

### 2.3. Data Analysis

Data were analyzed statistically using SAS version 9.4 (SAS Institute Inc., Cary, NC, USA). One-way analysis of variance (ANOVA) was used to verify differences in Fe concentration, RFeB%, and PA concentration among different meal models. The outcomes for the three variables (Fe concentration, RFeB%, and PA concentration) represented the three replicates of each sample. Fisher’s least significant difference (LSD) was calculated with the level of significance set at *p* < 0.05. Paired *t*-test analysis was used to assess differences in the five variables in the meal models featuring fortified vs. unfortified lentil. The associations among Fe concentration, RFeB%, and PA concentration were assessed using Pearson correlations at a *p* < 0.05 significance level [7]. Fe concentration, ferritin formation (ng ferritin/mg protein), RFeB%, PA, and PA:Fe molar ratio were compared to assess the effect of NaFeEDTA-fortified lentil (meal models 15 to 25) vs. unfortified lentil (meal models 1 to 11). A correlation analysis also was conducted for Fe concentration, PA concentration, and RFeB% to determine the relationships among these measures.

## 3. Results 

### 3.1. Fe Concentration, RFeB%, and PA Concentration 

**Figure 1:**
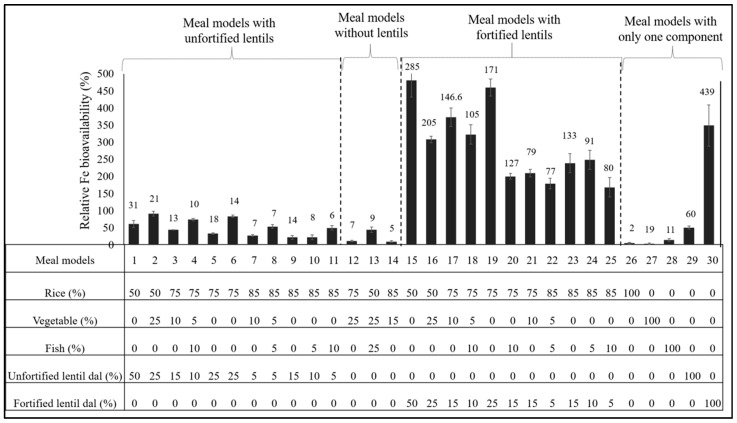
Relative iron bioavailability (RFeB%) and Fe concentration (μg g^−1^, above each bar) of 30 traditional Bangladeshi meal plan models containing unfortified lentil (meal models 1–11), no lentil (meal models 12–14), fortified lentil (meal models 15–25) and single components (meal models 26–30), assessed using a Caco-2 cell bioassay.

The average Fe concentration, RFeB%, and PA concentration of 30 meal model samples prepared with unfortified and fortified lentil are shown in Figure 1 and in Table S2. Significant differences were observed for Fe concentration, RFeB%, and PA concentration. The Fe concentration of the 30 meal plan models ranged from 2.1 µg g^−1^ (model 26; 100% rice) to 439.2 µg g^−1^ (model 30; 100% NaFeEDTA fortified lentil) and the PA concentration ranged from 1.2 mg g^−1^ (model 26; 100% rice) to 6.2 mg g^−1^ (model 29; 100% unfortified dal). RFeB% ranged from 3.7% (model 27; 100% vegetable) to 48.6% (model 15; 50% rice + 50% NaFeEDTA-fortified lentil); the control lentil had an RFeB% value of 30.9%. The highest Fe concentration, PA concentration, and RFeB% were found for meal models 30, 29, and 15, respectively. Among the 11 meal models (models 1 to 11) where unfortified lentil was used as a meal component (usage ranged from 5–50%, by weight), the highest Fe and PA concentrations were found in model 1, whereas the highest RFeB% was observed in model 2 (Figure 1). In meal models 15 to 25, where fortified lentil was used, the highest Fe and PA concentrations and RFeB% were observed in meal model 15 (Figure 1).

The iron concentrations for model 29 (100% unfortified lentil; Fe concentration 60 µg g^−1^) and model 30 (100% NaFeEDTA-fortified lentil; Fe concentration 439.2 µg g^−1^) indicate that lentil was the main component providing Fe across all of the meal plans (Figure 1). This also is reflected in the six models (12, 13, 14, 26, 27, 28) that contained no lentil and had low Fe concentrations (Figure 1) compared to models containing either fortified or unfortified lentil. Fish, vegetables, and rice did not notably affect Fe concentration as these components contain low amounts of Fe. The vegetable curry contained a higher amount of Fe (19.4 µg g^−1^) than did fish (11.4 µg g^−1^) or rice (2.1 µg g^−1^). The main component of meal models 2 to 14 and 16 to 25 was rice, ranging from 75 to 85%, by weight. Although the largest amounts of PA were found in unfortified lentil (6.2 mg g^−1^) followed by fortified lentil dal (4.6 mg g^−1^), the contribution of PA would have been mainly from rice, which comprised the major part of most meal models. For instance, meal models 9 and 23 had similar amounts of rice (85%) and lentil dal (15%), but the former contained unfortified dal and the latter, fortified dal. PA concentrations in meal models 9 and 23 were 2.4 and 1.7 mg g^−1^, respectively, of which 1.02 mg g^−1^ was contributed by rice.

Among the six meal models (1, 5, 9, 15, 19, 23) in which rice and lentil were the only ingredients, increasing the amount of rice generally decreased the Fe concentration, PA concentration, and RFeB%. The meal model that included rice (50%), fish (25%), vegetables (25%), and no lentil (model 13) contained a very low amount of Fe (8.7 µg g^−1^) but it was of higher relative bioavailability, which could be due to the low amount of PA in the meal. Models 4, 8, 18, and 22 contained similar amounts of vegetable (5%), but model 8 and 22 contained 10% more rice and 5% less fish and dal compared to models 4 and 18. This resulted in decreased Fe concentration, PA concentration, and RFeB%.

### 3.2. Comparison between Meal Models Containing Unfortified vs. Fortified Lentil

A comparison of Fe concentration, ferritin formation (ng ferritin/mg protein), relative Fe bioavailability (% of control lentil), PA concentration, and PA:Fe molar ratio between meal model groups featuring unfortified lentil (models 1 to 11) vs. fortified lentil (meal models 15 to 25) revealed significant differences for all parameters considered. Specifically, the average Fe concentration was significantly (*p* ≤ 0.001) higher in meal models with fortified lentil (136.2 µg g^−1^) compared to those with unfortified lentil (13.5 µg g^−1^). Ferritin formation (52.5 vs. 15.8 ng ferritin/mg protein) and RFeB% (290.0 vs. 51.2%) also were significantly (*p* < 0.001) higher in meal models with fortified lentil. PA concentration (2.1 vs. 2.4 mg g^−1^, *p* = 0.03) and PA:Fe molar ratio (1.5 vs. 16.9) were significantly (*p* ≤ 0.001) lower in meal models with fortified lentil.

### 3.3. Correlations between Measured Variables

Correlation coefficients between measured variables are presented in Table 1. Significant correlations were observed between Fe concentration and RFeB%, RFeB% and PA:Fe molar ratio, and Fe concentration and PA:Fe molar ratio when all meal models were considered. Significant correlations between Fe concentration and RFeB% as well as between RFeB% and PA:Fe molar ratio were observed for meal models with fortified lentil (models 15 to 25) but not unfortified lentil (models 1 to 11). Fe concentration and PA:Fe molar ratio had an inverse relationship for all meal models containing either unfortified or fortified lentil.

## 4. Discussion

Lentil fortification programs have been initiated with the aim of improving the Fe content in lentil because lentil serves as a major side dish in many countries, including Bangladesh. Due to poor absorption of intrinsic Fe from lentil, improvement in the Fe concentration in lentil dal and the increased absorption of Fe through fortification is a potential strategy to combat micronutrient malnutrition. In this study we assessed the bioavailability of Fe, using a Caco-2 cell bioassay, from a variety of traditional Bangladeshi meal models that contained either Fe-fortified or unfortified lentil.

In Bangladesh, the prevalence of anaemia in adolescent girls is ~30%, with iron deficiency considered the main cause [3]. Socioeconomic conditions also are reported to be a factor that, along with nutritional deficiency, influence dietary problems in rural Bangladeshi women, who consume lentil three (60%) or four (12%) times per week [28]. Lentil consumption is also increasing with the increasing price and reduced availability of animal protein. One study of the dietary habits of 384 rural women from northern Bangladesh revealed that 92% of respondents eat hotchpotch, a typical and traditional Bangladeshi dish with a pulse (usually lentil) and rice [28]. Thus, lentil fortification could be a potential approach to supplying a major part of the required amount of Fe to vulnerable people with Fe deficiency in Bangladesh.

Micronutrient bioavailability from fortified food depends on its absorption through the gastrointestinal tract for systemic utilization [29]. Bioavailability is the result of three major steps: digestibility (solubility of Fe in digesta), absorbability in the circulation system, and final processing and incorporation into a functional compartment of the body [30,31]. Different approaches, such as the chemical balance method, solubility or dialyzability, Caco-2 cell bioassay, hemoglobin repletion method, isotopic methods, and area under the curve for serum iron have been used to estimate non-heme iron absorption [32]. Other algorithms or combinations thereof have been used to assess Fe uptake based on Fe absorption from a single or complete meal [32]. In this study, a Caco-2 cell bioassay was used to measure Fe absorption. This model mimics conditions in the small intestine, and ferritin formation in the Caco-2 cell monolayers is considered as a marker for iron uptake [24]. Some limitations have been reported for the in vitro Caco-2 cell bioassay, for example, the in vitro model cannot fully mirror the human gut system that involves the effect of body Fe status and gut microflora on Fe uptake [24]. Considering these limitations, although this in vitro model is not a substitute for an in vivo model, it is a highly sensitive, cost-effective, and quick tool to measure Fe availability in foods [22,24]. Moreover, this model was found to be strongly correlated (R = 0.968, *p* < 0.001) with human Fe absorption studies [33], and with human and animal efficacy studies of Fe absorption from biofortified crops [34]. This model, therefore, can be considered to be thoroughly validated as a predictor of Fe absorption by humans. PA content was measured using a colorimetric assay kit, which is widely used as it gives accurate and reliable data, and saves cost and time [35]. Sometimes this kit gives a more accurate result than HPLC and quality controlling is easier than using HPLC if the person running the system is less experienced [36]. However, a limitation to the use of this kit is that it cannot measure *myo*-inositol in either its free or phytase/alkaline phosphatase released forms [35].

Iron absorption is influenced by both endogenous and exogenous factors [37]. The recipe used to prepare the various meal models used herein included different spices (turmeric, onion, garlic) and fat (canola oil). Bio-accessibility of Fe increased by 26.3% and 17.2% when 3.0 g of onion and 0.5 g of garlic, respectively, were cooked with 10 g of chickpea [38,39]. This could be due to the presence of sulfur-containing amino acids in *Allium* species that are reported to influence mineral status in animals. Moreover, spices also may contain phytic acid (inositol hexakisphosphate) and polyphenolic compounds (e.g., tannic acid and chlorogenic acid) [40]. The fortified and unfortified lentil used in the meal preparations are non-heme iron sources. Most polyphenols are located in the lentil seed coat, and the dehulled lentil used in this study would contain a low level of polyphenols, which would contribute to increased non-heme iron absorption in populations with limited Fe storage [41]. Turmeric is used extensively in countries of the Indian sub-continent, including Bangladesh. The most active constituent of turmeric is curcumin, a polyphenolic diketone. Curcumin forms a complex with solubilized Fe in aqueous solution with either Fe(II) or Fe(III) ion [42,43,44] and does not inhibit Fe absorption in young women [24]. Vegetables also contain significant amounts of vitamin A, carotenoids, and indigestible carbohydrates and the effect of these components on Fe absorption is unresolved [45]. Some vegetables used in this study to prepare vegetable curry, such as potato and sweet potato, contain a higher amount of Fe compared to the fish and the other vegetables used. This may explain the higher amount of Fe in vegetable (19.4 μg g^−1^; meal model 27) than in fish (11.4 μg g^−1^; meal model 28). A similar result also was found in another study conducted with traditional Bangladeshi meals [24].

Lentil consumption varies with age, gender, food habit, price, and availability of lentil in the market. The amount of vegetables in the meal models ranged from 5 to 25%, similar to traditional Bangladeshi meals. Fish comprised only 5 or 10% of the meals because the fish price in local markets is high and the consumption rate much lower than for other food items in the regular meal. Two meal models (models 3 and 17) are unique and represent hotchpotch, a ubiquitous meal for 1- to 5-year-olds and school-aged children in Bangladesh. In suburban areas of Bangladesh, “dal vaat” (rice and lentil or other pulses) is a common meal. Dried fish also is prevalent, and small amounts of dried fish with rice and lentil (models 6, 11, 20, and 25) also is a popular and widespread meal for local people in Bangladesh. The 30 meal models considered herein were designed with either unfortified or fortified lentil in varying amounts (5, 10, 15, 25, or 50%). Preliminary data (not shown) indicated that consumers prefer a thicker soup, which requires more lentil. This is favourable, as a higher amount of lentil dal in a meal will help to provide more of the required supply of Fe, and will increase the relative bioavailability.

The choice of NaFeEDTA-fortified lentil was based on the results of our two previous studies with respect to consumer acceptability [18,21]. Moreover, in the context of bioavailability, NaFeEDTA has proven to be more suitable than FeSO_4_ as a fortificant in legume-based flours [46]. In cowpea flour, higher PA:Fe molar ratios (3.0:1 to 3.3:1) are related to low iron absorption [46]. PA chelates with positively charged multivalent cations such as Fe, Zn, Mg, and Ca, forming insoluble complexes that precipitate in the neutral pH condition of the small intestine, thus decreasing Fe absorption [47]. In models 29 (100% unfortified lentil) and 30 (100% NaFeEDTA-fortified lentil), the PA content was 6.2 and 4.6 mg g^−1^ and the RFeB% was 50.6 and 349.2%, respectively (Table S2). These differences could be attributed to: (i) the higher Fe concentration in the NaFeEDTA-fortified lentil; (ii) the lower PA content in the NaFeEDTA-fortified lentil; or (iii) the fortification process, as dephytinization can inactivate phytates to a large extent [47].

In this study, PA concentration was assessed using a PA (total P) test kit (Megazyme International, Ireland). However, the concentration of polyphenolic components also could differ between fortified and unfortified lentil dal due to the effect of the fortification process. The PA concentration in the unfortified lentil meal (model 29) was significantly higher than in the fortified lentil dal meal (model 30). Thus, the PA:Fe molar ratio also was reduced from 8.8 in meal model 29 to 0.9 in meal model 30 (Table S2). This could be due to dephytinization during the fortification process. A previous study reported that for Fe-fortified fonio porridge, dephytinization and fortification reduced the PA:Fe molar ratio from 24:1 to 0.3:1 [48]. Again, a significant inverse correlation was found between RFeB% and the PA:Fe molar ratio. A similar result with respect to RFeB% and PA:Fe molar ratio was observed for meal models prepared with dehulled lentil and whole lentil [24].

Although no recommendations are in place for lentil fortification, the World Health Organization (WHO) has recommended some Fe fortificants and appropriate doses for fortification of wheat flour in 13 countries [49]. The Food and Agriculture Organizations of the United Nations/World Health Organization recommended nutrient intakes (RNIs) of Fe (mg) for females and males 19–50 years of age are 29.4 and 13.7 mg, respectively, based on 10% bioavailability [14]. In this study, the amount of fortified lentil ranged from 5–50% in meal models 15 to 25. These meal models feature the fortified lentil as part of the meal, and not as a supplement. The meal model with fortified lentil only (model 30; 100% NaFeEDTA-fortified lentil) can provide ~43.9 mg of Fe from 100 g of cooked dal (dry basis). This means that 100 g (dry basis) of meal model 19, which contains 25% fortified lentil, would contain ~11 mg of Fe. This could provide a major portion of the recommended nutrient intakes (RNIs) of Fe for adult males and females aged 19–50 (mentioned in [14]). Because the tolerable upper intake level of Fe for adults is 45 mg/day [50], the meal model with fortified lentil only (50 g person^−1^) is also safe for human consumption.

The study results showed that lentil was the major contributor of Fe and that the relative bioavailability of Fe increased when NaFeEDTA-fortified lentil was used in different meal models. Since different amounts of either fortified or unfortified lentil were used in different meal models, and the RNIs are advised on the basis of age, gender, pregnancy, and lactation period, recommendations for use of appropriate amounts of Fe-fortified lentil can be given for target populations. In this study, PA content was measured and considered to be the key inhibitor of Fe absorption. Since the PA concentration was significantly lower in the fortified lentil, it may be possible that levels of inhibitory polyphenols were also reduced in the fortified lentil, thereby increasing Fe absorption. However, it has been shown recently that not all polyphenols inhibit Fe absorption, and some have been identified as potential promoters of Fe uptake [51,52]. As we did not measure polyphenols in the meal models, our study cannot address this point.

## 5. Conclusions

Per capita global consumption of lentil is increasing rapidly. In some regions, however, the per capita consumption rate is actually decreasing due to higher demand. Fe-fortified lentil can provide a higher amount of Fe from a smaller amount of fortified lentil compared to unfortified lentil. This study demonstrated that lentil fortification is a promising and simple approach to help alleviate Fe deficiency, especially for countries in the developing world like Bangladesh, where most of the population consumes lentil in their daily meals.

## Figures and Tables

**Table 1:** Pearson correlation coefficients for iron (Fe) concentration vs. relative Fe bioavailability (RFeB%), bioavailability vs. phytic acid (PA):Fe molar ratio, and Fe concentration vs. PA:Fe molar ratio.

Meal Model	(Fe) vs. RFeB%	RFeB% vs. PA:Fe Molar Ratio	(Fe) vs. PA:Fe Molar Ratio
All (models 1 to 30)	0.832 **	−0.722 **	−0.627 **
(*n* = 30)	(<0.001)	(<0.001)	(<0.001)
Unfortified lentil (models 1 to 11)	−0.142	0.351	−0.628 *
(*n* = 11)	(0.685)	(0.299)	(0.0364)
Fortified lentil (model 15 to 25)	0.801 **	−0.763 **	−0.628 *
(*n* = 11)	(0.001)	(0.004)	(0.036)

** Correlation is significant at the 0.01 level (2-tailed); * Correlation is significant at the 0.05 level (2-tailed).

## Supplementary Materials

The following are available online at http://www.mdpi.com/2072-6643/10/3/354/s1. Table S1: Description of the 30 meal models, Table S2: Iron (Fe) concentration, relative Fe bioavailability (RFeB%), and phytic acid (PA) concentration (mean ± SD) and PA:Fe molar ratio of 30 meal plan models composed of varying percentages by volume of the amounts of rice, vegetable curry, fish and dal (lentil dish prepared with either fortified or unfortified lentil).
