# Supplementary material for: Relative Bioavailability of Iron in Bangladeshi Traditional Meals Prepared with Iron-Fortified Lentil Dal
Source: Nutrients. 2018 Mar 15;10(3):354. doi: 10.3390/nu10030354 (PMC5872772; doi:10.3390/nu10030354)
Supplement: Supplementary file 1 [file nutrients-10-00354-s001.zip › Rajib Nutrients-267861 Manuscript Supplementary table 1 and 2/Rajib Nutrients -267861 manuscript Supplementary table 2 Revised.docx]

**Table S2: Iron (Fe) concentration, relative Fe bioavailability (RFeB%), and phytic acid (PA) concentration (mean ± SD) and PA:Fe molar ratio of 30 meal plan models composed of varying percentages by volume of the amounts of rice, vegetable curry, fish and dal (lentil dish prepared with either fortified or unfortified lentil).**

|  | Rice (%) | Veg (%) | | Fish (%) | | Unfortified lentil dal (%) | | Fortified lentil dal (%) | | Fe  (μg g^-1^) | RFeB% | PA  (mg g^-1^) | PA:Fe molar ratio |
| --- | --- | --- | --- | --- | --- | --- | --- | --- | --- | --- | --- | --- | --- |
| Meal models with fortified lentil | | | | | | | | | | | | |  |
| Model 1 | 50 | | 0 | | 0 | | 50 | | 0 | 30.8 ± 0.6 | 61.0 ± 10.8 | 3.9 ± 0.0 | 10.6 |
| Model 2 | 50 | | 25 | | 0 | | 25 | | 0 | 20.9 ± 0.6 | 91.1 ± 6.9 | 3.1 ± 0.2 | 12.5 |
| Model 3 | 75 | | 10 | | 0 | | 15 | | 0 | 12.7 ± 0.9 | 44.7 ± 0.1 | 2.2 ± 0.1 | 14.4 |
| Model 4 | 75 | | 5 | | 10 | | 10 | | 0 | 9.8 ± 0.7 | 73.8 ± 3.1 | 2.2 ± 0.1 | 18.9 |
| Model 5 | 75 | | 0 | | 0 | | 25 | | 0 | 18.1 ± 0.1 | 33.5 ± 1.9 | 2.7 ± 0.2 | 12.8 |
| Model 6 | 75 | | 0 | | 10 | | 15 | | 0 | 14.4 ± 1.6 | 83.6 ± 3.8 | 2.6 ± 0.1 | 15.4 |
| Model 7 | 85 | | 10 | | 0 | | 5 | | 0 | 7.1 ± 0.2 | 27.4 ± 2.5 | 1.6 ± 0.1 | 19.7 |
| Model 8 | 85 | | 5 | | 5 | | 5 | | 0 | 7.5 ± 0.5 | 53.6 ± 5.3 | 1.9 ± 0.1 | 21.0 |
| Model 9 | 85 | | 0 | | 0 | | 15 | | 0 | 13.7 ± 1.3 | 22.0 ± 5.2 | 2.4 ± 0.2 | 14.6 |
| Model 10 | 85 | | 0 | | 5 | | 10 | | 0 | 8 ± 0.4 | 22.5 ± 6.94 | 1.6 ± 0.18 | 17.4 |
| Model 11 | 85 | | 0 | | 10 | | 5 | | 0 | 5.7 ± 0.4 | 49.8 ± 6.69 | 1.9 ± 0.03 | 28.3 |
| Meal models without lentil | | | | | | | | | | | | |  |
| Model 12 | 75 | | 25 | | 0 | | 0 | | 0 | 6.7 ± 0.3 | 11.4 ± 2.4 | 1.7 ± 0.1 | 21.1 |
| Model 13 | 50 | | 25 | | 25 | | 0 | | 0 | 8.7 ± 0.6 | 44.2 ± 8.3 | 2.2 ± 0.2 | 21.1 |
| Model 14 | 85 | | 15 | | 0 | | 0 | | 0 | 5.2 ± 0.1 | 9.6 ± 2.6 | 1.5 ± 0.0 | 24.8 |
| Meal models with fortified lentil | | | | | | | | | | | | |  |
| Model 15 | 50 | | 0 | | 0 | | 0 | | 50 | 285.2 ± 28.3 | 480.6 ± 48.8 | 2.8 ± 0.2 | 0.8 |
| Model 16 | 50 | | 25 | | 0 | | 0 | | 25 | 204.8 ± 34.4 | 308.0 ± 9.7 | 2.5 ± 0.1 | 1.0 |
| Model 17 | 75 | | 10 | | 0 | | 0 | | 15 | 146.6 ± 52.6 | 373.0 ± 26.8 | 1.9 ± 0.1 | 1.1 |
| Model 18 | 75 | | 5 | | 10 | | 0 | | 10 | 105 ± 25.1 | 322.7 ± 28.3 | 2.0 ± 0.0 | 1.6 |
| Model 19 | 75 | | 0 | | 0 | | 0 | | 25 | 170.9 ± 23.1 | 460.1 ± 25.1 | 2.2 ± 0.1 | 1.1 |
| Model 20 | 75 | | 0 | | 10 | | 0 | | 15 | 126.8 ± 28.3 | 200.4 ± 8.4 | 2.0 ± 0.0 | 1.3 |
| Model 21 | 75 | | 10 | | 0 | | 0 | | 15 | 78.8 ± 13.9 | 209.5 ± 10.8 | 1.8 ± 0.1 | 2.0 |
| Model 22 | 85 | | 5 | | 5 | | 0 | | 5 | 76.6 ± 10.8 | 179.0 ± 15.4 | 1.9 ± 0.1 | 2.1 |
| Model 23 | 85 | | 0 | | 0 | | 0 | | 15 | 132.8 ± 15.3 | 239.0 ± 27.8 | 1.7 ± 0.0 | 1.1 |
| Model 24 | 85 | | 0 | | 5 | | 0 | | 15 | 91.2 ± 7.1 | 248.8 ± 28.2 | 1.9 ± 0.1 | 1.7 |
| Model 25 | 85 | | 0 | | 10 | | 0 | | 5 | 79.8 ± 2.8 | 168.4 ± 28.1 | 2.0 ± 0.1 | 2.1 |
| Meal models, each contain one component at 100% | | | | | | | | | | | | |  |
| Model 26 | 100 | | 0 | | 0 | | 0 | | 0 | 2.1 ± 0.8 | 5.3 ± 2.5 | 1.2 ± 0.0 | 46.6 |
| Model 27 | 0 | | 100 | | 0 | | 0 | | 0 | 19.4 ± 0.5 | 3.7 ± 1.9 | 2.5 ± 0.1 | 10.8 |
| Model 28 | 0 | | 0 | | 100 | | 0 | | 0 | 11.4 ± 0.2 | 14.0 ± 4.0 | 1.4 ± 0.1 | 10.4 |
| Model 29 | 0 | | 0 | | 0 | | 100 | | 0 | 60 ± 0.6 | 50.6 ± 4.3 | 6.2 ± 0.1 | 8.8 |
| Model 30 | 0 | | 0 | | 0 | | 0 | | 100 | 439.2 ± 30.8 | 349.2 ± 60.4 | 4.6 ± 0.1 | 0.9 |
