# Supplementary material for: Relative Bioavailability of Iron in Bangladeshi Traditional Meals Prepared with Iron-Fortified Lentil Dal
Source: Nutrients. 2018 Mar 15;10(3):354. doi: 10.3390/nu10030354 (PMC5872772; doi:10.3390/nu10030354)
Supplement: Supplementary file 1 [file nutrients-10-00354-s001.zip › Rajib Nutrients-267861 Manuscript Supplementary table 1 and 2/Rajib Nutrients -267861 manuscript Supplementary table 1 Revised.docx]

**Table S1: Description of the 30 meal models.**

| Meal models | Rice (%) | Vegetable (%) | Fish (%) | Unfortified dal (%) | NaFeEDTA-fortified dal (%) |  |
| --- | --- | --- | --- | --- | --- | --- |
| Meal models with fortified lentil | | | | | | |
| Model 1 | 50 | 0 | 0 | 50 | 0 |  |
| Model 2 | 50 | 25 | 0 | 25 | 0 |  |
| Model 3 | 75 | 10 | 0 | 15 | 0 |  |
| Model 4 | 75 | 5 | 10 | 10 | 0 |  |
| Model 5 | 75 | 0 | 0 | 25 | 0 |  |
| Model 6 | 75 | 0 | 10 | 15 | 0 |  |
| Model 7 | 85 | 10 | 0 | 5 | 0 |  |
| Model 8 | 85 | 5 | 5 | 5 | 0 |  |
| Model 9 | 85 | 0 | 0 | 15 | 0 |  |
| Model 10 | 85 | 0 | 5 | 10 | 0 |  |
| Model 11 | 85 | 0 | 10 | 5 | 0 |  |
| Meal models without lentil | | | | | | |
| Model 12 | 75 | 25 | 0 | 0 | 0 |  |
| Model 13 | 50 | 25 | 25 | 0 | 0 |  |
| Model 14 | 85 | 15 | 0 | 0 | 0 |  |
| Meal models with fortified lentil | | | | | | |
| Model 15 | 50 | 0 | 0 | 0 | 50 |  |
| Model 16 | 50 | 25 | 0 | 0 | 25 |  |
| Model 17 | 75 | 10 | 0 | 0 | 15 |  |
| Model 18 | 75 | 5 | 10 | 0 | 10 |  |
| Model 19 | 75 | 0 | 0 | 0 | 25 |  |
| Model 20 | 75 | 0 | 10 | 0 | 15 |  |
| Model 21 | 75 | 10 | 0 | 0 | 15 |  |
| Model 22 | 85 | 5 | 5 | 0 | 5 |  |
| Model 23 | 85 | 0 | 0 | 0 | 15 |  |
| Model 24 | 85 | 0 | 5 | 0 | 10 |  |
| Model 25 | 85 | 0 | 10 | 0 | 5 |  |
| Meal models, each contain one component at 100% | | | | | | |
| Model 26 | 100 | 0 | 0 | 0 | 0 |  |
| Model 27 | 0 | 100 | 0 | 0 | 0 |  |
| Model 28 | 0 | 0 | 100 | 0 | 0 |  |
| Model 29 | 0 | 0 | 0 | 100 | 0 |  |
| Model 30 | 0 | 0 | 0 | 0 | 100 |  |
